# Down-staging (<pT2) of urothelial cancer at cystectomy after the diagnosis of detrusor muscle invasion (pT2) at diagnostic transurethral resection (TUR): is prediction possible?

**DOI:** 10.1007/s00428-012-1277-0

**Published:** 2012-07-10

**Authors:** Willemien Beukers, Titia Meijer, Cornelis J. Vissers, Joost L. Boormans, Ellen C. Zwarthoff, Geert J. L. H. van Leenders

**Affiliations:** 1 Department of Pathology, Josephine Nefkens Institute, Erasmus MC, PO Box 2040, 3000 CA Rotterdam, The Netherlands; 2 Department of Urology, Erasmus MC, Rotterdam, The Netherlands

**Keywords:** Cystectomy, Urothelial cell carcinoma, Down-staging, pT0

## Abstract

Urothelial cell carcinoma (UCC) with musculus detrusor (MD) invasion is treated by cystectomy. Subsequent pathologic evaluation of cystectomies does not reveal MD invasion (<pT2) in a subgroup of patients. Our objective was to identify features at diagnostic transurethral resection (TUR) predicting down-staging (<pT2) at cystectomy. Patients with pathologically confirmed MD invasion at TUR followed by cystectomy for UCC without (neo-) adjuvant therapy were included (*N* = 106). Slides of both TUR and cystectomy specimens were reviewed, and survival analyses were performed. In total, 27/106 (26 %) tumors were down-staged at cystectomy, of which 13 (12 %) had no residual tumor (pT0). There was no significant difference in age, gender, time interval between TUR and operation, number of slides sampled, and presence of TUR scar between down-staged (<pT2) and pT2 UCC. At review of TUR specimens (*N* = 52) with UCC initially diagnosed as pT2, MD invasion was not confirmed in eight cases (15 %). One case showed extensive histiocytic reaction misinterpreted as UCC; in four cases, muscularis mucosae had been considered MD, and in three cases, desmoplastic reaction mimicked MD. No histologic parameter at TUR was significantly associated with down-staging at cystectomy. Overall and disease-specific survival was not statistically different in down-staged and pT2 UCC. In conclusion, down-staging of UCC (<pT2) at cystectomy occurred in 26 %. At review of diagnostic TURs, MD invasion was not confirmed in 15 %. No clinical or pathologic parameter was predictive for down-staging at cystectomy. There was no difference in survival between down-staged and pT2-staged UCC.

## Introduction

Bladder cancer is the fourth most common cancer in males and ninth most common cancer in females [1]. The majority of bladder tumors primarily appear as non-muscle invasive disease (<pT2), whereas 20 % of urothelial carcinomas (UCCs) are infiltrating the musculus detrusor (MD) at presentation. Up to 25 % of initially non-muscle invasive tumors will eventually progress to MD invasive disease. Tumors limited to the mucosa (pTa/pT1) are conservatively treated, while bladder cancer infiltrating within or beyond the MD (≥pT2) requires aggressive treatment [2, 3]. After radical surgery, the 5-year recurrence free-survival of ≥pT2 UCC is only 56–58 % [4, 5]. Accurate pathologic staging of UCC at diagnostic transurethral resection (TUR) specimens is, therefore, an important parameter to determine the optimal therapy for individual patients.

Although accurate pathological staging at diagnostic TUR is pivotal for therapeutic decision-making, definite tumor stage can only be perceived after pathologic evaluation of cystectomy specimens. In a substantial number of patients, no invasion in the MD is identified at cystectomy, prompted by MD invasion at diagnostic TUR. These patients either show residual UCC without stromal invasion (pTa), UCC with invasion of the lamina propria (pT1), carcinoma in situ (CIS) (pTis), or no residual tumor at all (pT0). Down-staging of UCC to pT0 in cystectomy specimens is reported in 5 to 20 % [6–10]. Although down-staging is a well-known feature, it can be confronting for patient, urologist, and pathologist, raising the question whether the initial diagnosis prompting radical surgery was adequate.

Several pathologic features might mimic MD invasion of UCC. Infiltration of the muscularis mucosae (MM) and extensive desmoplastic stromal reaction can both be misinterpreted as MD invasion. Furthermore, it is not well-established whether UCC located directly adjacent to but not within the MD should be staged as pT2 disease. The objective of the present study was (a) to identify clinical and pathological features of UCC at diagnostic TUR, which correspond with down-staging at cystectomy and (b) to investigate the clinical outcome of patients with down-staged UCC after cystectomy.

## Material and methods

### Study population

In this study, we included patients with pathologically confirmed MD invasion at diagnostic TUR, followed by cystectomy for UCC without neo-adjuvant chemotherapy or radiotherapy. All patients were treated for UCC of the urinary bladder at Erasmus MC, location Dijkzigt, between June 1989 and May 2010. Patients were routinely followed up every 3 months on the first 2 years after cystectomy, semiannually on the third to fifth year, and subsequently at 12-month intervals. In case of progression, patients were followed up every 3 months. Loco-regional recurrent disease or distant metastases were assessed by CT of the abdomen and pelvis, chest X-rays, and bone scans (if indicated). Death was reported by treating physicians or general practitioner. Follow-up was defined as time from operation to event or last patient contact. Patients that deceased from other causes than bladder cancer, or that were lost to follow-up, were censored at the date of last contact. All clinical data were collected retrospectively from the electronic patient database.

### Pathologic review

Slides of cystectomies and diagnostic TUR specimens were retrieved from the archives and reviewed by a urogenital pathologist (GvL). Initial diagnoses had been made by a group of general clinical pathologists. At review, stage and grade were reassigned according the UICC 2002 TNM staging system and the WHO grading system 1973 and 2004, respectively. The following additional pathological parameters were recorded at review of the cystectomy specimens: number of slides of the urinary bladder sampled, presence of a TUR scar, and presence of CIS. At review of the diagnostic TUR specimens, the depth of invasion was established paying special attention to potential pitfalls such as MM invasion, extensive desmoplastic stromal reaction, invasion adjacent to but not within the MD, and invasion in between MD bundles. If classification was uncertain based on routine haematoxylin and eosin staining (HE), immunohistochemistry was performed. In case MD invasion was not obvious despite histology and additional immunohistochemistry, the tumor was classified as pT1 (*N* = 1), which is similar to our approach in daily practice, in which these tumors are reported as “pT1 at least.” The use of the samples for research purposes was approved by the Erasmus MC Medical Ethics Committee according to the Medical Research Involving Human Subjects Act (MEC-2004-261).

### Immunohistochemistry

Formalin-fixed, paraffin-embedded tissue sections (5 μm) from TUR specimens were cut and mounted on aminoacetylsilane-coated slides (Starfrost, Berlin, Germany). Sections were deparaffinised in EZprep (Roche Diagnostics, Indianapolis, IN, USA). For all antibodies, heat-induced epitope retrieval was performed in CC1 buffer (high pH) (Roche Diagnostics) for 64 min. The slides were incubated with monoclonal Desmin antibody (clone DE-R-11; 1:50; Novocastra, Newcastle, UK) or monoclonal keratin 7 antibody (clone OV-TL 12/30; 1:1,000; Biogenex, San Ramon, CA, USA) at 36 °C for 32 min, followed by amplification using UltraView Universal DAB detection kit (Roche Diagnostics). All reactions were performed in an automatic BenchMark Ultra device (Roche Diagnostics). Finally, the slides were counterstained, dehydrated, cleared in xylene, and mounted in Pertex (Histolab Products, Gothenburg, Sweden).

### Statistical analysis

For comparison of continuous and categorical parameters of the pT2 and the down-staged group at cystectomy, Mann–Whitney and Fisher's exact test were performed, respectively. Kaplan–Meier curves with log-rank analysis were used for comparison of survival. A *p* value <0.05 was considered statistically significant. All analyses were performed using the Predictive Analytics Software 17 (SPSS Inc., Chicago, IL, USA).

## Results

### Patient characteristics

Between May 1989 and June 2010, a total of 160 patients had undergone cystectomy for a urothelial malignancy at Erasmus MC, comprising of 135 (84.4 %) males and 25 (15.6 %) females (Table 1). The mean age of the patients at operation was 62.4 years (range 33.2–82.5 years). The majority of male patients had undergone a radical cystoprostatectomy (*N* = 128; 94.8 %), which was combined by a left (*N* = 4), right (*N* = 4), or bilateral (*N* = 1) nephro-ureterectomy. Four (3.0 %) male patients had been treated by simple cystectomy, of whom three had undergone a previous radical prostatectomy for prostate adenocarcinoma. Three men (2.2 %) had been treated by partial cystectomy. A total of 17 (68 %) female patients had undergone a cysto-hysterectomy with adnexa extirpation, combined with a left (*N* = 1) or right (*N* = 1) nephro-ureterectomy. Eight (32 %) females had been treated with simple cystectomy (Table 1).

**Table 1:** Clinical characteristics of 160 patients operated for urothelial cell carcinoma

*N* = 160	No. of patients (%)
Age (median; range)	62.4 (33.2–82.5)
Sex	
Male	135 (84)
Female	25 (16)
Type of operation	
Male	
Radical cystoprostatectomy	128 (95)
With nephro-ureterectomy	9
Simple cystectomy	4 (3)
Partical cystectomy	3 (2)
Female	
Cysto-hysterectomy with adnexa extirpation	17 (68)
With nephro-ureterectomy	2
Simple cystectomy	8 (32)
Indication for cystectomy	
Musculus detrusor invasion	137 (86)
Therapy-resistant recurrent pTa/pT1 or CIS	17 (11)
Involvement of the prostatic urethra by UCC/CIS	4 (2)
Very large superficial high grade UCC	1 (0.5)
Urothelial CIS combined with sigmoid adenocarcinoma	1 (0.5)

*UCC* urothelial cell carcinoma, *HG* high grade, *CIS* carcinoma in situ

The clinical indication for cystectomy was UCC with MD invasion (≥cT2) in 137 patients (85.7 %), therapy-resistant recurrence of superficial high grade pTa/pT1 UCC and/or CIS (*N* = 17; 10.6 %), involvement of the prostatic urethra by superficial high grade UCC/CIS (*N* = 4; 2.5 %), a very large superficial UCC (pTa; *N* = 1; 0.6 %), and urothelial CIS combined with sigmoid adenocarcinoma with potential involvement of the urinary bladder (*N* = 1; 0.6 %). Of the 137 patients who had been operated for MD invasion, 23 (16.8 %) patients had undergone neo-adjuvant therapy and 8 (5.8 %) had clinical MD invasion without pathological confirmation. For further analysis, we included the remaining 106 (77.4 %) patients who had pathologically confirmed MD invasion (≥pT2) at diagnostic TUR, followed by cystectomy without neo-adjuvant therapy.

### Characterization of down-staged UCC

Of the 106 patients with ≥pT2 UCC at TUR, histologic evaluation of the cystectomy specimen revealed 13 cases (12.3 %) without residual tumor (pT0), 5 (4.7 %) with CIS only, 3 (2.8 %) with pTa, 6 (5.7 %) with pT1, 33 (31.1 %) with pT2, 37 (34.9 %) with pT3, and 9 (8.5 %) with pT4 disease. Therefore, 27 (25.5 %) cases had lower stage UCC at cystectomy, then at initial diagnostic TUR, which showed ≥pT2 disease (Table 2). No statistically significant difference in age (*p* = 0.64), gender (*p* = 1.00), or number of days between diagnostic TUR and operation (*p* = 0.96) existed between down-staged (<pT2) and concordantly staged (≥pT2) cystectomies (Table 3).

**Table 2:** Tumor characteristics of 106 patients with ≥pT2 on diagnostic transurethral resection (TUR), followed by cystectomy for urothelial cell carcinoma without (neo-) adjuvant therapy

*N* = 106	No. of patients (%)
Stage	
pT0	13 (12)
pTis	5 (5)
pTa	3 (3)
pT1	6 (6)
pT2	33 (31)
>pT2	46 (43)
Concomitant CIS	16
Grade	
WHO 1973	
No tumor	13 (12)
G1	0 (0)
G2	9 (9)
G3	79 (79)
WHO 2004	
No tumor	13 (12)
PUNLMP	0 (0)
LG	0 (0)
HG	88 (88)

*CIS* carcinoma in situ, *PUNLMP* papillary urothelial neoplasm of low malignant potential, *LG* low grade, *HG* high grade

**Table 3:** Clinical (*N* = 106) and pathologic (*N* = 58) characteristics of down-staged (<pT2) and ≥pT2 urothelial carcinoma at cystectomy

No.	Clinico-pathologic variable	Down-staged <pT2	≥pT2	*p* value
106	Age (median; range)	64.9 (35.1–73.9)	64.1 (33.2–82.5)	0.64
	Male/female (%)	23:4 (85/15)	68:11 (86/14)	1.00
	Time after TUR (days) (median; range)	57 (15–113)	55 (12–220)	0.96
58	Number slides (median; range)	10 (3–33)	10 (2–23)	0.62
	TUR reaction (%)	20/27 (74)	17/31 (55)	0.13

Review of pathologic cystectomy characteristics was performed only on the down-staged (<pT2) and pT2 urothelial cancers

*TUR* transurethral resection

To investigate whether down-staging was due to difference in sampling, we histologically reviewed all cystectomy slides of ≤pT2 UCC. Stages pT3 and pT4 were not evaluated (*N* = 46). In two cases, slides were not available for review, leaving a total of 58 cases for analysis. The mean number of slides sampled from the urinary bladder was ten in both the down-staged and the concordantly staged pT2 group (*p* = 0.62). Histological reaction to previous TUR was found in 20/27 (74 %) cases of the down-staged group as compared to 17/31 (55 %) cases in the pT2 group (*p* = 0.13), indicating that under-sampling of the cystectomy specimen was not the cause of tumor down-staging after operation (Table 3).

**Fig. 1:**
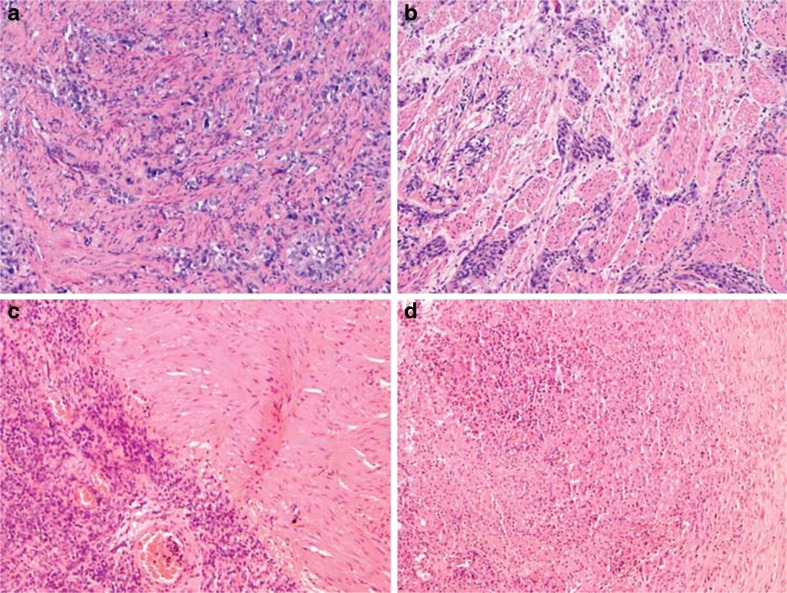
Assessment of urothelial cell carcinoma invasion in relation to the musculus detrusor. **a**, **b** Urothelial carcinoma invading in between musculus detrusor fascicles. **c** Urothelial cancer located adjacent to but no in between musculus detrusor fascicles. **d** Histiocytic reaction after previous diagnostic transurethral resection, misinterpreted as urothelial cancer invading the musculus detrusor. HE; original magnifications ×100

**Fig. 2:**
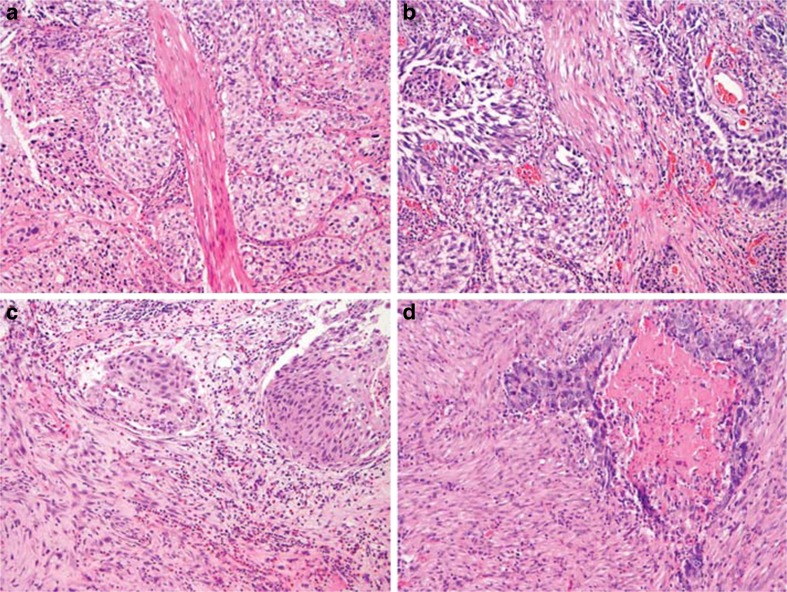
Pitfalls of urothelial cell carcinoma invasion of musculus detrusor. **a**, **b** Muscularis mucosae fascicles mimicking musculus detrusor. **c**, **d** Extensive desmoplastic stromal reaction misinterpreted as musculus detrusor invasion. HE; original magnifications ×100

**Fig. 3:**
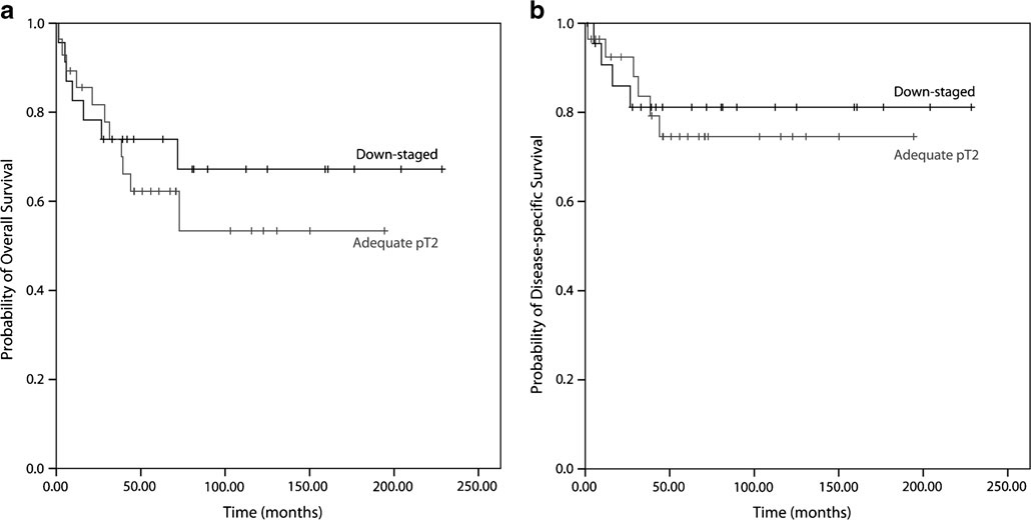
Survival analysis of down-staged and pT2-staged urothelial cell carcinoma after cystectomy. There was no difference in overall **a** (*p* = 0.53) or disease-specific **b** (*p* = 0.76) survival of patients with <pT2 versus pT2 urothelial cancer at cystectomy

In order to find histological features at diagnostic TUR that corresponded with down-staging at cystectomy, we reviewed all diagnostic TUR slides of the 58 patients with ≤pT2 UCC. Six cases were not available for review, leaving 52 cases for further analysis. At review, one case showed extensive histiocytic infiltration while no UCC was found in the diagnostic TUR specimen, which was verified with negative immunohistochemical Ker7 staining (Fig. 1). This case was incorrectly diagnosed as UCC at the time of diagnosis; subsequent cystoprostatectomy indeed did not reveal UCC (pT0). In the remaining 51 cases, grade according to WHO 1973 (*p* = 0.28) or 2004 (*p* = 0.26), and concomitant CIS (*p* = 0.55) were not associated with down-staging at cystectomy. Of the tumors, 40/51 (78.4 %) demonstrated invasion into the MD, 4 (7.8 %) showed tumor adjacent to the MD but not in between the muscle fascicles, and 7 (13.5 %) did not reveal invasion up to MD bundles convincingly (Fig. 2). Of these seven cases, four (7.7 %) showed invasion in between small smooth muscle fascicles, which were not characteristic for MD, but could represent MM and three (5.8 %) demonstrated extensive desmoplastic stromal reaction without tumor infiltration into the MD. The desmoplastic stromal reaction was probably interpreted as MD invasion at initial diagnosis (Fig. 3). In four cases, Desmin immunohistochemistry expression was used to facilitate identification of smooth muscle fascicles at review, in case of cauterization or differentiation with desmoplastic stromal reaction. There was no significant relation of the type of tumor invasion in the TUR specimen and the subsequent findings at cystectomy (Table 4).

**Table 4:** Pathologic characteristics of urothelial cell carcinoma at diagnostic transurethral resections (TUR) in the down-staged (*N* = 21) and adequately pT2-staged group (*N* = 30)

*N* = 51	Down-staged	Adequate pT2	*p* value
	*N* (%)	*N* (%)	
G2/G3	2/19 (10/90)	7/23 (23/77)	0.28
LG/HG	0/21 (0/100)	3/27 (10/90)	0.26
Concomitant CIS	8 (38)	9 (30)	0.55
Desmoplastic stromal reaction	0 (0)	3 (100)	N/A
MM invasion	2 (50)	2 (50)	1.00
Adjacent to MD	2 (50)	2 (50)	1.00
In between MD fascicles	17 (43)	23 (57)	1.00

*LG* low grade, *HG* high grade, *CIS* carcinoma in situ, *MM* muscularis mucosae, *MD* musculus detrusor

### Survival analysis

The mean follow-up of the pT2 group and the down-staged group was 61.6 and 70.9 months, respectively. To investigate whether patients who were down-staged at cystectomy had a favorable outcome versus those with stage pT2 at cystectomy, survival analysis was performed. We excluded 9/60 (15 %) patients with positive regional lymph nodes from the analysis, i.e., 4/27 (14.8 %) in the down-staged group and 5/33 (15.2 %) in the pT2 group. There was no significant difference in overall (*p* = 0.53) and disease-specific (*p* = 0.76) survival between the adequately staged and down-staged group (Fig. 3).

## Discussion

In a significant number of cases, urologists and pathologists are confronted with UCC down-staged (<pT2) at cystectomy, although operation was prompted by an MD invasive tumor at diagnostic TUR. Down-staging at cystectomy might have several causes, such as successful neo-adjuvant therapy, complete UCC resection at TUR, or false-positive diagnosis at TUR. In the present study, we investigated the incidence and the possible origin of down-staging in a well-defined cohort of 106 cystectomy patients, of whom operation was prompted by pathologic MD invasion at diagnostic TUR and who had not undergone neo-adjuvant therapy.

We found down-staging at cystectomy in 26 % of the study population. In 12 %, no residual UCC (pT0) was identified, which is in line with numbers (6–20 %) reported by others [6–9]. Age, gender, and interval between TUR and operation were not statistically different between concordantly staged pT2 and the down-staged group. Since the number of slides sampled at grossing and frequency of identifying a TUR scar at microscopic evaluation of cystectomy specimens was similar in both groups, down-staging was not due to sampling error.

At review of the diagnostic TUR specimens, in 12 out of 52 (23.1 %) cases, UCC infiltration into the MD could not be confirmed. Discrepancy with the original TUR staging was caused by false interpretation of (a) histiocytic reaction as invasive UCC (1.9 %), (b) invasion of the MM as MD invasion (7.7 %), (c) extensive desmoplastic stromal reaction as MD (5.8 %), and (d) UCC impinging upon the MD as invasion into the MD (7.7 %). As far as we know, the latter subtle distinction has not been subject of study yet. If UCC adjacent to MD would be classified as pT2 disease, as is often done in pathologic practice, the prevalence of down-staging UCC at review of TUR specimens was 15.4 % (8/52). Interestingly, this percentage of staging discordance is in line with the percentage of original pT1 UCC up-staged to pT2 disease at review, indicating that inter-observer variability is also existing in assessing MD invasion [11]. Distinguishing between MM and MD invasion at diagnostic TUR is a well-known challenge in surgical pathology, which is hampered by problems in tissue orientation and potential MM hyperplasia. In addition, MD-infiltrating tumors may partially destruct muscle fibers, leaving thin residual MD bundles resembling MM [12]. Despite these difficulties, accurate recognition of MD invasion is crucial for patient treatment according to the current guidelines. Immunohistochemical expression of the cytoskeletal protein Smoothelin is reported to be stronger in MD than in MM [13–15]. Therefore, additional immunohistochemistry using Smoothelin can be useful in differentiating both muscle layers. While variable Smoothelin expression patterns can be appreciated when both layers are present in the same specimen, its value in classifying a limited amount of smooth muscle fibers in small specimens remains to be established. Therefore, we decided not to include this marker in the current study. Finally, extensive desmoplastic myofibroblastic reaction evoked by UCC invasion might histologically mimic MD [16]. In this case, immunohistochemical detection of Desmin is helpful to distinguish myofibroblasts from MD smooth muscle cells [17].

No statistically significant difference in overall and disease-specific survival was found between the down-staged and concordantly staged pT2 group. Interestingly, the percentage of patients with positive lymph nodes was similar between the two groups as well. Regarding the patients with pT0 disease, survival after cystectomy was not different from the patients with higher tumor stages. This is in accordance with the findings of Thrasher et al. [18]. This implicates that MD invasive UCC at diagnostic TUR with down-staging at cystectomy should still be regarded as pT2 disease. The clinical significance of pT0 disease at cystectomy is, however, still under debate. Volkmer et al. excluded patients with neo-adjuvant therapy and found a significantly better disease-specific survival in patients with pT0 disease at cystectomy versus patients with maximal pT2a disease at cystectomy [19]. Likewise, Tilki et al. investigated a group of 4,430 patients treated with radical cystectomy and found significantly better clinical outcomes in patients with pT0 disease than those with clinical and pathological stage T2 [20]. These results are conflicting with the findings in our study, which could be explained by the fact that in our study a relatively small number of patients were included.

This relatively low number of patients is a limitation of this study and was due to our strict selection criteria, i.e., exclusion of non-urothelial cancer such as squamous carcinoma and adenocarcinoma, presence of neo-adjuvant therapy, availability of slides for review, and omitting high-stage disease (pT3/4) from analysis. Nevertheless, to the best of our knowledge, this is the first study specifically investigating the nature of down-staging in UCC, in which we retrospectively demonstrate 15 % inter-observer variability in the diagnosis of clinically decisive MD invasion. For practical purposes, important lessons can be drawn from this study. First, UCC invasion directly adjacent to MD bundles can be classified as pT2 disease at TUR since down-staging in this group did not occur more frequently than in UCC with invasion into the MD fascicles. Secondly, the presence of pT2 disease at cystectomy in patients with MM invasion or extensive desmoplastic stromal reaction yet no MD invasion at TUR as well as the significant inter-observer variability of T1-2 staging raises the question whether the current clinico-pathologic guidelines for treatment of invasive UCC can be optimized. Herr performed a re-staging TUR in patients with histologically proven pT2 disease [21]. If re-staging revealed pT0 or pT1 disease, patients were offered immediate cystectomy or careful follow-up. Interestingly, the 10-year disease-specific survival was similar in both groups with significantly better survival in patients with pT0 as compared to pT1 tumor at re-staging. Since cystectomy is a surgical procedure with 90-day complication rates as high as 46 % and peri-operative mortality ranging from 1 to 4 % [3, 22], further studies on the use of re-TUR and imaging modalities for the treatment and follow-up as alternative for radical operation in a subgroup of UCC patients are mandatory.

In conclusion, we have shown that 26 % of patients with initial pT2 stage at diagnostic TUR were down-staged at subsequent cystectomy. No clinical or pathologic parameters could be identified that were associated with down-staging (<pT2) of UCC at cystectomy. Review of diagnostic TUR specimens revealed inter-observer variability, with pT2 to <pT2 in 15 % of the cases. No difference in survival was found in patients with down-staged versus pT2 UCC at cystectomy.
